# Supplementary material for: Are carbon emissions trading and green financial instruments synergistic? -Comprehensive quantitative research based on content analysis
Source: PLoS One. 2024 Mar 7;19(3):e0298601. doi: 10.1371/journal.pone.0298601 (PMC10919601; doi:10.1371/journal.pone.0298601)
Supplement: S1 Data — (ZIP) [file pone.0298601.s001.zip › Data/地级市Z_F工作报告.zip!/╣π╢1⁄2/╔ε█┌╩╨/└┤╫╘╣1⁄2╓┌║┼í╛┬φ┐╦╩2╛▌═°í┐_2014.docx]

各位代表：

　　现在，我代表深圳市人民政府向大会作政府工作报告，请予审议，并请各位政协委员和其他列席人员提出意见。

　　一、2013年工作回顾

　　2013年是深圳经济特区全面贯彻落实党的十八大和习近平总书记视察广东深圳等一系列重要讲话精神的开局之年，是我市有效应对错综复杂的外部形势，打造深圳质量取得显著成效的一年。在党中央国务院、省委省政府和市委的坚强领导下，我们紧紧围绕“三个定位、两个率先”的总目标，坚持质量引领、创新驱动，着力深化改革、扩大开放，加强社会建设、民生改善，在科学发展中实现新跨越，在质量提升中增创新优势，完成了市五届人大五次会议确定的主要目标任务。

　　初步预计，全年本市生产总值增长10.5%左右；固定资产投资2501亿元，增长14%；社会消费品零售总额4433.6亿元，增长10.6%；进出口总额5373.6亿美元，增长15.1%，居内地大中城市首位；全口径公共财政收入4818亿元，其中地方公共财政预算收入1731亿元，增长16.8%；居民消费价格涨幅2.7%。

　　（一）质量型增长特征更加鲜明。深圳质量理念贯穿于经济社会发展各领域和全过程，质量第一成为各行各业的共同追求，深圳荣获全国首个“质量强市示范市”称号。经济指标实现“双突破”，人均GDP突破2万美元，出口总额突破3000亿美元。资源能源消耗实现“双下降”，用水总量下降3300万立方米，预计万元GDP能耗下降4.3%。经济效益实现“双提高”，地方公共财政预算收入增速提高6.2个百分点，规模以上工业企业利润增速提高20个百分点。需求结构实现“双优化”，服务业投资占投资比重超过8成，工业产品内销比重首次超过5成。城市功能实现“双跃升”，深圳港集装箱吞吐量跃居全球第三，机场旅客吞吐量跃升至3000万人次以上。

　　（二）创新驱动发展动力更加强劲。深入实施创新驱动发展战略，全面落实市科技创新大会精神，着力完善综合创新生态体系，不断提升自主创新能力，加快建设国家创新型城市。创新型经济持续发力。全社会研发投入占GDP比重提高到4%，提前达到“十二五”规划目标。华为公司成为我国首个跻身全球研发支出50强企业。战略性新兴产业总规模达1.63万亿元，增长19.8%，对经济增长贡献率超过50%。下一代信息网络、高端生物医学工程、生物基因产业纳入国家战略性新兴产业集聚发展试点，国际生物谷总体发展规划出台。新认定国家高新技术企业591家。技术创新向引领式迈进。新增国家、省、市级重点实验室、工程实验室、工程中心、技术中心等176家，累计达955家。组织重大技术攻关项目66项。新组建3D显示、大数据等产学研资联盟。PCT国际专利申请量超过1万件，占全国48.1%。继获国家技术发明一等奖、科技进步一等奖等10个奖项之后，又有9项科研成果获国家科技大奖；腾讯、比亚迪等公司4项发明获中国专利金奖，占全国1/5。第四代移动通信TD－LTE技术国际领先，深圳企业获得LTE商用合同数全球第一。开放式创新步伐加快。引进10个海外高层次创新团队，三星通信设备研究院等研发机构落户深圳。新增开展境外研发业务企业31家，华大基因成功收购全球知名的美国CGI公司。高交会、文博会、国际人才交流大会、电子信息博览会、IT领袖峰会等影响力不断扩大，国际创新中心建设取得新进展。

　　（三）产业转型升级步入正轨。科学把握转型升级的方向、重点、力度和时序，在战略性新兴产业快速发展的基础上，抢先布局未来产业，出台生命健康、海洋经济、航空航天等产业规划及政策，加快构建梯次型现代产业体系，打造可持续的产业竞争力。产业结构更趋合理。预计二三产业结构调整为43.4∶56.6，服务业比重再提高近1个百分点，其中金融业增加值突破2000亿元，本外币存款余额跃居全国第三，新引进法人金融机构26家。产业向价值链高端延伸。先进制造业占规模以上工业比重71.3%。华星光电成为全球第五大液晶电视面板厂商，长安标致雪铁龙汽车项目正式投产。加工贸易企业中委托设计和自有品牌比重超过65%。成功举办首届中国（深圳）国际工业设计大展，工业设计产业增速为工业增速3倍以上。荣获全国唯一“中国钟表之都”称号，黄金珠宝产业集聚基地成为全国知名品牌创建示范区，水贝?中国珠宝指数正式运行，服装、家具、眼镜等产业品牌化、时尚化转型加快。深圳位居2013福布斯中国商业城市榜第二位。企业结构进一步优化。4家本土企业入选世界500强，27家企业进入财富中国500强。引进菜鸟网络等总部企业20家，总部企业数量在全球370个新兴市场城市中列17位。新增规模以上法人企业3667家。出台支持中小微企业健康发展的若干措施，小微企业贷款余额增长20.3%。淘汰清理转型低端落后企业3145家。新兴业态快速发展。电子商务交易额突破9500亿元，“深商e天下”电子商务平台集聚企业超过3000家，深圳成为首批信息消费试点城市。三网融合产业规模突破3000亿元。网络支付、借贷和股权众筹等互联网金融发展居全国前列。

　　（四）绿色低碳成为城市新特质。全面推进生态文明建设，着力提升大气、水环境质量和绿化水平，建设宜居宜业的绿色家园。市民绿色福利持续提升。PM2.5处于内地副省级以上城市最低水平。新建改造公园80个。大鹏半岛国家地质公园建成开放。投放公共自行车1万辆。完成124公里、3.56万亩生态景观林带建设。盐田区成为国家水土保持生态文明区，光明新区成为国家绿色生态示范城区。龙岗河、坪山河、观澜河等河流水质持续改善。新增污水管网218公里，527个单位（小区）开展垃圾减量分类试点。节能减排成效显著。万元GDP水耗三年累计下降33.4%，完成“十二五”规划目标的167%，成为全国节水型示范城市。预计化学需氧量三年累计减排2.55万吨，提前完成“十二五”减排任务，二氧化硫、氨氮分别累计减排5100吨和3600吨，完成“十二五”减排任务80%和96%，氮氧化物完成年度减排目标。全面实施绿色建筑标准，新增绿色建筑380万平方米、节能建筑1000万平方米，全年可节电14.8亿度。全面推广使用国IV柴油；淘汰黄标车近3万辆，是上年的1.5倍。绿色低碳影响力不断扩大。率先启动碳排放权交易，占全市碳排放总量40%的机构上线交易，成为全国交易量最大的碳市场。累计推广应用新能源汽车6363辆，居全球城市前列。国际低碳城建设加快，引进太空科技南方中心等10个重点项目，成功举办首届国际低碳城论坛。深圳成为中欧城镇化伙伴关系合作城市。

　　（五）改革开放取得新突破。坚持改革不停顿、开放不止步，出台全面深化改革三年总体方案和年度改革计划，重点领域和关键环节改革取得新成效。前海开发开放实现大提速。27个基础设施建设项目全面开工。产业准入目录出台，现代服务业集聚发展态势强劲，入区企业超过3500家。前海股权交易中心等7家新型要素交易平台设立运行。跨境人民币贷款业务率先开展，备案金额超过150亿元。外商投资股权投资企业试点扎实推进。深港青年创新创业基地启动建设。前海商事法庭、国际仲裁院、廉政监督局成立运作。市场活力进一步激发。率先全面实施商事登记制度改革，改革以来新增商事主体数同比增长152%。优化国有资本布局，市属国有企业利润总额增长17.4%。深入推进土地管理制度改革，建立了市场化的产业用地供需平台，首宗原农村产业用地入市交易。实行以备案为主的社会投资管理改革，社会投资占比提高到80.8%。营业税改征增值税范围扩大，20万户试点纳税人总体税负减轻34.4%，全市纳税人共减税81.6亿元。社区股份合作公司改革试点启动。民生领域改革力度加大。公立医院“管办分离”改革取得突破，公立医院管理中心正式成立运作。出台鼓励社会资本举办三级医院若干规定，内地首家香港独资眼科医院正式运营，社会医疗机构诊疗量占总量的23.3%。义务教育阶段公办学校实施积分入学。开放合作水平不断提升。实际使用外资54.7亿美元。累计设立境外企业和机构2537家，对外直接投资约占全省的47.7%；深圳企业新签对外工程承包合同金额231.9亿美元，约占全国地方企业的22.5%。出台实施保税区转型升级总体方案。深港跨界基础设施建设加快，深圳河治理四期工程开工，莲塘/香园围口岸前期工作完成。深莞惠区域协调发展总体规划实施。深汕特别合作区引进华润云计算数据中心等重大项目。全方位对口帮扶河源、汕尾工作启动，新一轮扶贫开发“双到”及重点县帮扶顺利开展。援疆、援藏和对口帮扶贵州等取得新成效。

　　（六）现代化国际化城市功能进一步增强。以国际一流城市为标杆，全面提升城市发展质量，强化中心城市服务辐射功能。城市空间开发格局不断优化。特区一体化新三年计划实施，原特区外投资占全市比重达73%。调整优化基本生态控制线，高标准建设3万亩基本农田，完成土地整备18平方公里，开工天安岗头等49个城市更新项目，深圳再获省“三旧改造”一等奖，福田区成为全国国土资源节约集约模范区。现代化基础设施更加完善。深圳机场新航站楼启用，转场圆满成功。空港国际航线客流量增长25.6%，海港国际班轮航线达238条。厦深铁路投入运营，广深沿江高速、博深高速、清平高速二期建成通车，文锦渡口岸新旅检大楼投入使用，轨道交通三期7、9、11号线加快建设，丹梓西路等34条断头路打通，80个片区交通综合改善工程完成。新增管道天然气居民用户11万户。深圳北站综合交通枢纽等14项工程获6个国家优质工程奖、6个詹天佑奖和2个鲁班奖。智慧城市建设深入推进。4G网络正式商用运营，下一代互联网规模化商用启动，互联网、家庭宽带、无线宽带覆盖率分别达84.2%、83.1%和87.7%，蝉联国内“最互联网城市”称号。新增光纤入户30万户，开展30个智慧社区试点。深圳成为首个政务信息共享国家示范市，城市管理和民生服务信息加快实现“统一采集、部门共享、联网运行”。国际化城市形象持续提升。新增圣彼得堡、雅典、赫尔辛基、明斯克等10个国际友好交流城市，国际友城和友好交流城市达54个。荣获联合国教科文组织“全球全民阅读典范城市”称号，《人文颂》在联合国教科文组织总部成功演出。WTA国际女子网球公开赛、深圳国际马拉松赛、中国杯帆船赛、世界模特和精英模特全球总决赛等高水平赛事成功举办。旅游总收入增长13.8%。城市公共安全体系建设加强。发布城市公共安全白皮书，出台台风暴雨灾害公众防御指引，扎实开展危险边坡、危化品、泥头车等隐患排查治理，着力提高安全水平。加强食品药品安全监管，食品、药品分别抽检27987和1315批次，依法查处其中不合格品1255和10批次，分散、落后的旧屠宰场全部关闭。推进平安深圳建设，立体防控体系不断完善，110接报刑事治安警情数继续下降，社会治安进一步好转。

　　（七）保障和改善民生力度加大。坚持尽力而为、量力而行，保基本、可持续，加快民生幸福城市建设，111项民生实事完成109项，还有2项正加快推进。基本民生保障水平不断提升。全市财政对九类重点民生领域投入达1061亿元，比重提高2.6个百分点。预计居民人均可支配收入达44650元。新增就业人数超过8万人，城镇居民登记失业率2.35%。社保基金收支分别增长22.7%、21.7%。保障性住房开工1.7万套、竣工2.21万套、供应2.7万套，建立安居型商品房、公共租赁住房轮候机制，安排人才安居货币补贴10亿元，人才住房和补贴惠及约20万人。优质饮用水入户工程启动。社会事业发展步伐加快。新增幼儿园学位2万个、中小学公办学位2.04万个。建成普惠性幼儿园503所，学前儿童健康成长补贴惠及21.5万名儿童。统一公办学校生均拨款和设备设施配备标准。发放民办义务教育学位补贴、民办中小学教师长期从教津贴5.8亿元。南方科技大学新校园启用，香港中文大学（深圳）启动校区工程完工。累计6所特色学院开始筹建。市儿童医院住院大楼等一批重大卫生项目建成启用，新增病床1042张。家庭医生签约户数累计达23.2万户。疾病预防控制水平进一步提升。出台加快老龄服务事业和产业发展的意见以及养老设施专项规划、城市无障碍改造规划，新增养老床位766张和老年人日间照料中心10家，市养老护理院、社会福利中心新址项目开工。深圳籍运动员在第12届全运会上获18金19银的历史最好成绩。国防动员和双拥工作不断加强，妇女儿童、残疾人事业等取得新进步。社会管理创新扎实推进。社会组织达6944家，增长23%，专业社工5900多人。志愿者之城建设加快，注册志愿者超过90万。广泛开展各类慈善募捐活动，成功举办第二届中国慈展会。加快“织网工程”和“风景林工程”建设，新建社区服务中心150家、社区家园网409个，罗湖区、坪山新区成为全国社区治理和服务创新实验区。社会信用体系建设加强，市企业信用信息系统与金融业统一征信平台实现数据共享、联动监管。文化建设取得新成就。积极弘扬社会主义核心价值观，举办读书月、外来青工文体节等各类文化活动1.5万场，公共文化服务体系更加健全，市民文明素质进一步提升。当代艺术馆与城市规划展览馆工程加快推进，深圳书城宝安城动工建设。创意信息港成为国家对外文化贸易基地，《城市节奏》等5部作品获第十届中国艺术节群星奖，福田区入选创建国家公共文化服务体系示范区。

　　（八）政府自身建设不断加强。深入开展党的群众路线教育实践活动，推动政府工作提质提效，法治政府和服务型政府建设取得新成效。切实转变工作作风。严格落实中央八项规定，坚决反对“四风”，市政府下发公文同比减少30.3%，各类简报减少50%，全市性会议申报核减率83%，“三公”经费下降11.5%以上。加强廉政建设，着力规范权力运行和政府行为。健全常态化的深入基层调研机制，全年专项调研服务企业1500多家，一批影响企业发展的问题得到切实解决。加快建设法治政府。严格遵守和执行各项法律法规，自觉在人大的法律监督和工作监督、政协的民主监督下开展工作，定期向市人大报告、向市政协通报工作，起草并向市人大常委会提交立法案10部，积极回应人大代表、政协委员对社会热点问题的关注，办理市人大代表建议709件、市政协提案535件。废止、修改规章20项和规范性文件249件。加强政府门户网站、政务微博、政务微信等平台建设，推进政务信息公开。着力提高行政效能。出台行政审批事项目录管理办法，实现审批事项法定化、规范化。全面推进部门内部审批流程再造，审批时限进一步压缩。建成广东省网上办事大厅深圳分厅，市区两级网上办事大厅全部开通。完善政府绩效管理，试行绩效面谈制度。出台行政电子监察工作规定，审批业务提前办结率进一步提高。此外，信访、统计、计生、司法、民族、宗教、港澳、对台、侨务、口岸、民防、档案工作服务水平持续提升。

　　各位代表，过去一年，我们在党的十八大、十八届二中、三中全会和习近平总书记一系列重要讲话精神的指引下，紧紧依靠全市人民，坚定不移打造深圳质量，全力以赴推动有质量的稳定增长和可持续的全面发展，以创新理念赢得发展先机，以抢先布局获取竞争主动，在改革开放中实现了动力、活力全面增强，在转型升级中实现了质量、速度同步提升，在协调发展中实现了生态、民生持续改善。这些成绩的取得，主要得益于我们坚决贯彻落实中央、省和市委的各项决策部署，坚持深圳质量理念不动摇，以质量促进稳定增长，以质量创造竞争新优势，开辟发展新空间；得益于我们坚持稳中求进，着力把握调结构与稳增长的平衡点，在更高水平上推进经济发展方式转变，在主动转型中防控经济失速风险，在创新发展中打破要素成本制约，增强了长远发展后劲；得益于我们始终坚持社会主义市场经济改革方向，大胆探索、先行先试，推进综合配套改革，着力释放改革红利，为可持续的全面发展注入强劲动力；得益于全市人民团结一致、奋力拼搏，以劳动创造幸福生活，以实干成就美好梦想，形成了加快科学发展、共建美丽深圳的强大合力。在这里，我代表深圳市人民政府，向在各个岗位上辛勤工作的全市人民，向给予政府工作大力支持的人大代表和政协委员，向各民主党派、工商联、各人民团体和社会各界人士，表示崇高的敬意！向中央和省各部门、中央和各省区市驻深单位，向驻深解放军、武警官兵，向关心和支持深圳经济特区发展的港澳同胞、台湾同胞、海外侨胞和国际友人，表示衷心的感谢！

　　质量追求永无止境，科学发展任重道远。在前进的道路上，我们面临着外部不确定性因素较多、区域竞争加剧、资源环境约束趋紧等一系列严峻挑战。同时，城市发展和政府工作还存在一些亟待解决的问题。一是发展中不平衡、不协调的问题依然存在，破解二元结构、加快特区一体化的任务还很艰巨；二是城市人口规模大、结构不合理，统筹人口与经济社会协调发展的难度加大；三是安全生产、食品安全等存在薄弱环节，城市公共安全保障能力有待进一步提高；四是汽车尾气排放、部分河段污染等问题比较突出，灰霾天数在总体下降中还有反复，生态环境建设还需要付出更大努力；五是教育、医疗、文化等领域优质资源总量不足、布局不均，不能很好地满足群众日益增长的公共服务需求；六是政府管理中干预过多、监管不力并存，一些部门内部审批流程复杂，转变政府职能、提升行政效能还需作出更大努力。我们要直面挑战，正视问题，积极采取措施，认真加以解决。

　　二、2014年工作思路和重点

　　2014年是认真贯彻落实党的十八届三中全会精神的第一年，也是完成本届政府各项目标任务的冲刺之年。新的一年，世界经济不稳定不确定因素依然存在，刺激政策力度和效应逐步减弱，新的增长动力源尚不明朗，但总体上缓慢复苏态势基本确立。国内经济运行还存在下行压力，部分行业产能过剩、宏观债务水平上升等突出问题尚未缓解，但稳中有进、稳中向好的基础进一步巩固。面对新形势新任务，我们要有强烈的忧患意识和机遇意识，在新一轮全面深化改革的历史征程中，以义不容辞的责任担当和奋发有为的工作激情，抢抓机遇、开拓进取、先行先试，想干敢干快干会干，以改革开放、质量引领、创新驱动的主动，赢得科学发展的主动和国际竞争的主动。

　　今年政府工作的指导思想是：全面贯彻落实党的十八大、十八届二中、三中全会和习近平总书记一系列重要讲话精神，坚持稳中求进工作总基调，坚持深圳质量理念不动摇，牢牢把握市场化、法治化、国际化改革发展方向，以前海开发开放为突破口，把改革创新贯穿于经济社会发展各领域全过程，以改革促发展、促创新、促转型、促开放、惠民生，努力实现有质量的稳定增长、可持续的全面发展，推动现代化国际化先进城市建设再上新台阶。

　　以改革促发展，就是坚持把改革作为推动发展的根本动力，把握发展规律、创新发展理念、破解发展难题，以市场化释放发展活力，以法治化提升治理能力，以国际化增强城市竞争力，在持续深化改革中保持特区领先优势，推动实现更长时期更高质量的发展。

　　以改革促创新，就是顺应世界科技革命新趋势和创新创意创造新特点，改革科技管理体制，优化创新资源配置，完善创新组织方式，构建更加开放协同的综合创新生态体系，让创新要素加快集聚、创新成果加速转化、创新活力竞相迸发，推动创新向前沿高端迈进，不断增强质量型增长、内涵式发展的创新驱动力。

　　以改革促转型，就是把市场的决定性作用和政府的主动作为更好结合起来，强化市场利益导向，完善考核评价体系，加快构建有效的激励约束机制，使转型升级的方向与提升质量的目标相一致，结构调整的力度与稳定增长的要求相协调，以更低的成本实现经济结构持续优化和发展质量全面提升。

　　以改革促开放，就是主动适应经济全球化新形势，积极为开放创造更好的体制环境，加快完善与国际接轨的规则体系，打造扩大开放的战略平台，推动货物贸易服务贸易相促进、引进来走出去相结合、对内对外开放相协调，构建高水平、高质量的开放型经济新体系。

　　以改革惠民生，就是以改革的红利增进民生的福利，着力深化以保障和改善民生为重点的社会事业改革，推进社会治理体制创新，提高公共服务多元供给能力，加快基本公共服务均等化，强化社会政策托底功能，促进社会和谐有序，不断提升市民生活质量，让改革发展成果更多更好惠及全体市民。

　　今年经济社会发展的主要预期目标是：本市生产总值增长10%左右；万元GDP能耗下降4.25%，万元GDP水耗下降6%；居民人均可支配收入与经济增长保持基本同步，城镇居民登记失业率控制在3%以内，居民消费价格涨幅控制在3.5%左右。这里需要说明，10%左右的增速，是建立在质量提升前提下的速度，是在更高发展水平上稳中求进的速度，既综合了世界经济温和复苏、国内经济稳中向好、深化改革释放活力、新的增长点开始发力等积极因素，也充分考虑了各种潜在风险和挑战。实现这一目标，需要我们保持足够的定力和自信，更需要全市上下顽强拼搏，付出艰苦努力。

　　今年重点做好十个方面工作：

　　（一）着力推进十大改革，创造体制机制新红利。深圳经济特区因改革而生，因改革而兴，因改革而强。我们要着眼于市场在资源配置中起决定性作用，紧紧围绕“三化一平台”，在过去率先建立社会主义市场经济体制的基础上，继续敢闯敢试、先行先试，全面落实深化改革实施意见和年度改革计划，努力在重点领域和关键环节取得新突破，率先形成有利于质量型发展的体制机制。一是营造更加公平竞争的市场环境。探索建立负面清单制度，放宽市场准入，着力消除对非公有制经济的各种隐性壁垒，支持非公有制企业进入特许经营领域，不断优化营商环境。深化商事登记制度改革，依托信用体系，完善监管制度，实现宽进严管。推进资源性产品价格改革。深化以管资本为主的国资国企改革。进一步提升混合所有制经济水平。推进社区股份合作公司改革试点，加快政企、社企分开。二是深入推进金融创新发展。落实全面深化金融改革创新的若干意见，加快航空航运、稀贵金属等新型要素交易平台组建运行。率先开展巨灾保险试点，创新保险资金运用方式。鼓励民间资本设立或并购金融机构，积极培育一批民营金融标杆企业。出台互联网金融政策，大力发展互联网金融等创新业态。三是加快建立现代财政制度。完善政府全口径预算管理体系，改进预算管理制度，探索建立跨年度预算平衡机制，继续推进预算公开。改革财政专项资金使用方式，加强绩效评估。盘活财政存量资金，启动国库现金管理改革。规范政府性债务管理。四是深化土地管理制度改革。优化土地空间规划体系，推进节约集约用地。完善土地产权制度，构建土地二次开发利益共享机制，扩大原农村集体经济组织经营性用地入市范围，探索完善高度城市化地区土地可持续利用机制。五是改革科技管理体制。创新科技计划管理方式，优化评审程序与组织方式。改革研发资金投入方式，引入创投机制，发挥财政资金引导和杠杆作用，撬动更多资源投向科技创新。六是健全公共服务多元供给机制。大力支持社会力量参与教育、医疗、养老等社会事业发展。加快国家教育领域综合改革试点和医药卫生综合改革，创建国家养老服务业综合改革试点区。改革社保业务办理方式，推进资源共享、全市通办，为市民提供更加便捷的服务。七是深化文化体制机制改革。推进市属国有文化集团公司制改造。鼓励民营文化企业参与国有文化单位和企业改制重组、合作经营。完善文化产业投融资和交易平台。引入竞争机制，推动公共文化服务社会化发展。培育外向型文化企业，支持文化企业到境外开拓市场。八是创新社会治理方式。完善有效预防和化解社会矛盾体制，健全重大决策社会稳定风险评估机制。实行网上受理信访制度，把涉法涉诉信访纳入法治轨道。探索优化行政区划设置，进一步理顺街道与社区管理事权，创新社区服务管理。推进街道城市管理执法权相对集中，减少权责交叉、多重执法。九是推进生态文明制度建设。探索建立自然资源资产产权制度和用途管制制度。完善生态控制线管理，实施最严格的源头保护制度。健全碳交易市场运行和监管体系，扩大交易覆盖面，推动跨区域碳市场合作和碳金融产品开发。探索推进节能量、排污权、水权交易市场建设。十是更加积极主动推进政府改革。进一步简政放权，编制政府部门权责清单和运行流程图，推进行政审批事项“减、放、转”，凡是市场机制能有效调节的经济活动一律取消审批。优化部门内部流程和职能配置，加快并联审批和跨部门协同办理，建成网上办事大厅，提高审批效率和服务水平。深化大部门制改革，理顺委局关系，形成决策科学、执行坚决、监督有力的运行机制，完善食品药品和市场监管、自然资源监管、安全生产监管体制。优化法治政府建设指标体系，健全政府规章和规范性文件动态清理、适时调整机制，依法规范和约束行政权力运行。加强政府绩效考核，强化结果运用，完善激励约束机制。深化公务员分类及聘任制改革。推进事业单位去行政化，探索实施以事定费，开展事业单位人事制度综合配套改革试点。加大政府购买公共服务力度，编制并公开政府职能转移和购买服务目录。开展公务用车制度改革。

　　（二）落实国家战略，加快建设前海开放发展新平台。前海开发开放是国家新时期改革开放的重大举措。要按照“依托香港、服务内地、面向世界”的要求，发扬敢为天下先的精神，落实好比特区还要特的先行先试政策，高质量推动前海开发开放全方位突破，建设深圳质量典范区。一是率先营造国际一流营商环境。加快建设社会主义法治示范区，探索建立与国际接轨的民商事法律机制，推动设立前海商事法院，打造更高水平的国际化商事仲裁平台。完善前海法定机构运作机制，最大限度减少行政审批，在企业设立、经营许可、人才引进等方面提供高效优质的一站式服务。积极推动深港货物贸易便利化和服务贸易自由化，推进深港职业资格互认，促进各类要素更加便捷流动。二是全力推进现代服务业开放发展。抓住人民币国际化和利率市场化的重大机遇，积极在跨境金融、离岸金融、创新金融等领域先行先试，引进一批创新型金融机构和要素交易平台，加快建设跨境人民币业务创新试验区，努力成为我国金融业对外开放的试验示范窗口。扩大跨境人民币贷款规模，力争备案金额超过500亿元。加大全球投资推广力度，突出高端定位、总部优先、业态先进，引进一批国际知名的现代服务业企业，实现入区企业超过1万家，服务业规模突破1000亿元。三是全面掀起前海开发建设高潮。瞄准世界最佳商务区，加快基础设施和重点项目建设，新开工建筑面积400万平方米以上，总投资规模1200亿元，形成开工一批、建设一批、竣工一批的大开发格局。创新深港联合推进机制，健全深港两地高层定期沟通会晤和常态化工作联络机制，吸引更多香港企业参与前海开发建设。

　　（三）聚焦湾区经济，构建区域协同发展新优势。发达的湾区经济是世界一流滨海城市的显著特征。深圳毗邻港澳、背靠珠三角、地处亚太主航道，要紧紧抓住我国实施海洋强国战略、建设21世纪海上丝绸之路及世界经济重心东移等重大机遇，发挥经济繁荣、市场开放、创新活跃、改革领先的综合优势，加快构建开放型经济体系，拓展经济纵深，联手打造产业发达、功能强大、开放互动、区域协同的湾区经济，在更大范围、更高层次参与全球经济竞争合作，实现更高质量、更高能级的发展。一是优化湾区经济发展布局。紧紧围绕陆海资源统筹、产业集群发展，高标准加快前海深港现代服务业合作区、深圳湾总部基地、大鹏湾国际生态旅游区、国际生物谷等建设，不断强化福田、罗湖、南山商务中心功能，着力打造盐田、宝安、坪山中心区和大空港、北站商务区、光明门户区、大运新城等新的区域经济增长极，形成片区协调互动的湾区经济发展形态。二是增强湾区经济核心功能。依托深圳亚洲最大陆路口岸和海港、空港、信息港三港联动优势，着力增强航运、贸易、金融集聚辐射功能。加强与东盟国家基础设施互联互通，吸引国际合作机构落户，鼓励企业到东盟国家投资，打造21世纪海上丝绸之路枢纽城市。完善国际集装箱班轮航线网络，进一步提高国际中转比例，培育发展邮轮经济、游艇产业等高端航运服务业，提高港口经济附加值。提升新航站楼综合服务功能，推动空港经济发展。积极发展国际会展业和供应链管理、跨境电子商务等新型业态。加快金融中心城市建设，集聚更多金融高端资源，全年新引进法人金融机构20家以上。三是提升湾区经济国际影响力。加快转变外贸发展方式，在输出产品的同时，更加注重输出资本、技术、管理、服务和品牌，服务贸易出口达到490亿美元。提高利用外资质量，实现利用外资58亿美元。全面落实国际化城市建设行动纲要，加强国际语言环境、国际化社区和国际学校建设，瞄准国际知名城市拓展友城网络，全方位开展国际交流合作。四是推动湾区经济协同发展。面向亚太，立足全国，依托泛珠三角，加强与周边地区协作，开拓更加广阔的经济腹地，提升湾区经济发展整体水平。加快广深港客运专线、莲塘/香园围口岸、深圳河治理四期等跨界基础设施建设，推进落马洲河套等边境地区合作开发。深入落实CEPA、ECFA和粤港服务贸易自由化政策，加强与香港、澳门、台湾地区交流合作，推动区域资源高效配置、市场深度融合。推进珠三角一体化发展，加强深莞惠合作，推动深茂铁路、深中通道、穗莞深城际轨道、赣深高铁等项目的规划建设，打通区域合作战略通道，形成湾区经济发展联动效应。

　　（四）坚持质量优先，打造产业升级新优势。以质量创造价值，以质量赢得市场，是产业长远可持续发展的关键。要牢牢把握质量导向，在更高层次上加快产业转型升级，促进经济发展全面提质增效。一是突出增量优质、存量优化，完善梯次型现代产业体系。巩固提升战略性新兴产业领先优势，加快留仙洞、大沙河创新走廊等产业基地集聚区开发建设。全面落实未来产业规划和政策，加快培育海洋经济、航空航天、生命健康等产业。大力发展工业设计，推动服装、钟表、家具、眼镜、黄金珠宝等产业转型发展。推进保税区、出口加工区转型升级。落实总部经济政策和支持大型骨干企业加快发展的若干措施，引进一批总部企业。继续淘汰落后产能。二是强化内需拉动，进一步释放消费和投资潜力。主动顺应消费需求升级，培育发展信息、健康、文化和时尚消费等新业态，加快建设国家信息消费试点市和电子商务示范城市，全市商品销售总额突破2万亿元。抢抓新型城镇化重大机遇，积极开拓内需市场，进一步提高工业产品内销比重。提高投资质量、扩大投资规模，加快推进华星光电二期、腾讯滨海大厦、平安国际金融中心等重大产业项目建设。三是打造深圳标准，铸就深圳品牌。以国际先进水平为标杆，全面提升深圳产品食品质量标准，以更高标准创品牌、树信誉、拓市场，保障消费安全，增强产品竞争力。强化标准实施的基础支撑，完善检验检测服务体系，使深圳成为重要的产品认证和检测中心。加大产品质量检测力度和频次，强化标准实施监督检查，把企业执行标准情况纳入信用记录，健全守信激励和失信惩戒联动机制，创建全国诚信建设示范城市。

　　（五）加快创新驱动，强化经济内生增长新优势。创新是实现有质量、可持续发展的必然选择。要瞄准前沿、聚焦重点，更加注重原始创新和协同创新，努力争创国家自主创新示范区。一是加强创新基础能力建设。建成国家基因库一期工程，加快太空科技南方中心等项目建设，争取未来网络实验室等国家重大科技基础设施布局深圳。推进超材料工业技术、下一代高速大容量光传输技术、高通量基因测序及组学技术等国家级创新载体建设。提升国家、省、市级重点实验室、工程实验室、工程中心、技术中心等建设发展质量，创新载体总数超过1000家。强化重大科技基础设施和创新载体开放运行、资源共享，更好地发挥公共技术平台对创新的支撑作用。二是实施科技攻关计划。组织实施50个以上重大技术攻关项目，在云计算、大数据、移动互联网、生命信息、新能源等重点领域，实现产业共性技术、关键技术研发与应用突破，形成一批高质量的核心自主知识产权和国际、国家技术标准。三是加大人才引进培养力度。开工建设特色学院集中校园，加快筹建若干所特色学院。完善高层次专业人才队伍建设系列政策，继续实施“孔雀计划”，引进10个海外高层次创新团队。加快技能人才队伍建设。完善人才安居政策。四是优化综合创新生态体系。推动技术、产业、金融、管理、商业模式创新跨界融合，促进创新多主体协同、多要素联动、多领域合作。建立一批产学研资用联盟，探索更具弹性和活力的组织形式。加快科技金融服务体系建设，促进股权投资产业发展，支持中小企业在主板、创业板上市，在新三板等场外市场挂牌。设立中小微企业发展基金，加速创新成果产业化、资本化。深入推进国家知识产权和商标战略实施示范城市建设，全面提升知识产权保护和运营水平。利用信息化手段，为分布式、网络化创新和全社会“微创新”构建开放式创新平台。

　　（六）突出绿色低碳，增创宜居宜业环境新优势。优良的生态环境是生存之本、发展之基和民生之需。要加快出台生态文明建设规划，实施最严格的源头保护、总量控制、损害赔偿和责任追究制度，提升环境质量，建设美丽深圳。今年安排节能环保资金增长35.8%。一是加强水环境综合治理。深入开展鹏城水更清行动，着力改善前海湾、深圳湾等近海水环境，加快茅洲河、龙岗河、坪山河、葵涌河等综合整治，推进观澜河“一河两岸”景观提升工程。投入运营公明污水处理厂，推进沙井、燕川污水处理厂二期、东涌污水处理厂和老虎坑、上洋等污泥处理厂建设。建成污水管网200公里。二是改善大气环境质量。全面实施大气环境质量提升行动，推广使用国V汽油，加快新能源汽车、货运行业LNG车辆和港口岸电应用。扩大黄标车限行范围，全部淘汰非专业类公务黄标车。完成妈湾电厂深度脱硫和除尘改造，推进垃圾焚烧厂烟气脱硝，加强施工扬尘管理。提升工业燃烧源治理水平，完成300家重点排放企业废气治理。三是提升绿化水平。推进区域绿地和生态廊道建设，构建“四带六廊”自然生态安全体系。加快大鹏半岛、田头山等自然保护区建设，建成187公里、3.89万亩生态景观林带。开展立体绿化示范，新建改造提升公园50个。强化绿道管理，完善配套设施。扎实推进垃圾减量分类试点。四是开展绿色低碳合作。出台国际低碳城总体规划及专项规划，加快核心启动区项目建设，引进一批龙头企业，办好第二届国际低碳城论坛。大力发展节能环保产业。健全区域环保合作机制，加强环保信息共享和跨界污染协调治理，提高区域环境污染突发事件应急处置能力。

　　（七）注重完善功能，推进有质量的城市化。落实中央城镇化工作会议精神，坚持以人为本、优化布局、完善功能、协调发展，提高城市建设质量和精细化管理水平。一是提高空间资源利用效率。构建适应存量土地开发利用的规划管理制度体系，有序推进土地整备和城市更新，存量用地占供地总量达68%以上，提升土地节约集约利用水平。加快后海、华为科技城等重点片区综合开发，推进岗厦、笋岗—清水河、白石洲和盐田三村、四村等重点城市更新项目建设，推动插花地城市更新。加快解决城市化历史遗留问题。二是深入实施特区一体化建设新三年计划。今年市本级投资73.8%安排在原特区外，加快基础设施、城市管理、环境保护一体化和教育、医疗、文化等基本公共服务均等化，不断完善原特区外城市功能。着力改善梅林关、南头关、布吉关等二线关交通环境，建成开通彩田路北延段，启动梅观高速市政化改造，开工建设坂银通道等一体化发展重点项目。三是完善城市交通体系。着力优化城市路网，建成坂澜大道，推进东部过境高速、坪盐通道、外环高速、南坪快速三期、桂庙路快速化改造等工程。加快完善盐田港后方陆域路网和推进疏港道路建设。积极建设公交都市，推进轨道交通三期和龙华现代有轨电车示范线规划建设，优化新增60条以上公交线路，公交分担率提高到55.5%。落实城市交通白皮书，强化需求管理，提升智能化管理水平，加大交通拥堵综合治理力度，打通24条断头路，实施路边停车收费管理试点。四是打造国际一流信息港。全面落实加快信息化发展若干意见，推进“百兆到户、千兆到企”，光纤入户数增加到150万。加快无线城市建设，无线宽带覆盖率达到88%，基本实现重要公益性公共场所WIFI免费全覆盖。深化三网融合试点，扩大广电、电信业务双向开放。实施交通、水电、三防减灾等基础设施智慧化改造。五是增强城市保障能力。加快抽水蓄能电站、迭福LNG项目、分布式能源示范工程、输变电工程建设。加大引水工程和水库建设保护力度，推进公明供水调蓄、清林径引水调蓄等工程建设。新开工一批小区优质饮用水入户工程。加强粮油等重要商品储备管理，继续抓好菜篮子工程建设。新建市政燃气管道110公里，新增管道天然气居民用户8万户。

　　（八）实施重大民生工程，提高民生幸福水平。政府一切工作的根本目的就是以民为本、服务民生。要坚持以解决问题为导向，以群众利益为依归，着力把经济发展成果更多惠及民生，提升民生发展质量。今年市财政安排重点民生领域支出增长23.5%。一是启动实施12项重大民生工程。着眼长远、系统谋划，集中启动实施食品药品安全、蓝天工程、基础教育、名医、文体惠民、安居、养老、社区公园建设、污水管网建设、茅洲河治理、消防能力提升、地面坍塌防治等12项跨年度、跨“十二五”规划的重大民生工程，总投入超过1000亿元，力争通过几年持续努力，集中解决一批民生领域的突出问题，实现民生幸福水平和城市安全保障能力显著提升。二是扎实办好116件年度民生实事。围绕市民关心的切身利益问题，按照可量化、可操作、可考核的原则，年度安排116件具体民生实事，当年实施、当年见效，通过一个个项目的落实、一件件实事的办理，让市民不断得到更多实惠。三是完善可持续的社会保障体系。出台基本公共服务均等化行动计划。坚持积极的就业政策，推动创业孵化基地建设，以创业带动就业，力争全年新增就业8万人，保持零就业家庭动态归零。最低生活保障标准和最低工资标准分别提高到每人每月620元和1808元。孤儿最低养育标准总体提高15%。构建租售补相结合、多层次广覆盖的住房保障体系，新开工保障性住房2.5万套、竣工2.5万套、供应2.8万套。落实企业年金、职业年金个人所得税递延纳税政策，探索社会保障市场化补充机制，推进重大疾病补充医疗保险。完善社会救助体系，发展社会福利事业，加快市养老护理院等项目建设，新增公办养老床位1500张，老年人日间照料中心10家以上。四是提升教育事业发展质量。优化资源投入和师资配备，加快教育信息化，促进基础教育优质均衡发展。出台提升学生综合素养的指导意见，启动第二批百校扶百校和特色学校创建工作。新改扩建公办中小学校28所，建成盐田高级中学，新增普通高中学位4200个、公办义务教育学位3.7万个。加快7所高级中学规划建设。新增普惠性幼儿园100所和幼儿园学位2万个。创新发展高等教育，推动香港中文大学（深圳）正式招生和哈工大深圳研究生院开展本科教育，加快深圳大学西丽校区等项目建设。开展全民素质提升试点，加强职业教育与培训，完善终身教育体系，建设学习型城市。五是提高医疗卫生事业发展水平。着力引进国内外名医、名院、名诊所等优质医疗资源，为市民提供更高质量的医疗卫生服务。推动国家癌症中心深圳医院建设，规划建设新一代卫生医疗重大基础设施。建成市第二人民医院综合大楼、北大深圳医院外科住院楼和宝荷医院，加快新安、新明、聚龙等医院建设，新增病床2400张。提升社康中心医疗配置和服务水平，推广家庭医生服务，加大全科医师引进培养力度。发展网络医疗，推动公立医院医学检验、影像检查结果互联互认，完善预约诊疗、电子病历等服务，更加方便市民就医。依法落实单独两孩政策。完善重大流行性疾病防控机制。六是大力推进文化体育等社会事业发展。着力构建现代公共文化服务体系，积极打造图书馆之城、钢琴之城、阅读之城，不断提升设计之都影响力，创作更多人民群众喜闻乐见的文化精品。办好国际钢琴协奏曲比赛、深圳读书月、市民文化大讲堂等重大文化活动。促进哲学社会科学繁荣发展。加强社会主义精神文明建设和道德教育，探索建立城市荣誉制度。加快当代艺术馆与城市规划展览馆、大鹏所城二期等文化设施建设，加强南头古城等历史文化遗产的保护与利用。发展体育运动，完善体育设施，推进公共体育场馆、学校体育设施向社会免费或优惠开放，广泛开展全民健身运动，增强市民体质。大力引进国际高水平赛事，办好WTA和ATP国际网球公开赛、中国杯帆船赛、国际马拉松赛、高尔夫欧巡赛等重大赛事。发展公益慈善事业，办好第三届慈展会。做好民族宗教工作。加强国防动员体系建设，积极创建新一轮全国、全省“双拥模范城”。推进深汕特别合作区建设，扎实开展对河源、汕尾扶贫开发“双到”及重点县帮扶工作。以喀什深圳城和深圳产业园为重点，积极做好援疆工作。继续抓好援藏、帮扶贵州等工作。

　　（九）创新社会治理，激发社会发展活力。充分发挥政府主导作用，构建机制科学、内容丰富、参与多元的治理体系，努力保障市民安居乐业、社会安定有序。一是充分发挥社会组织作用。鼓励支持社会组织参与社会治理和公共服务，加快培育发展行业协会商会、公益慈善、社区服务等各类社会组织，推动完善社会组织相关法规、内部治理结构和跨部门综合监管体系。提升志愿服务专业化、信息化、社会化水平，探索建立专业社工职业体系。支持工会、共青团、妇联、残联等群团组织开展活动。二是强化基层管理服务。深入推进社会建设“织网工程”和“风景林工程”。整合社区管理资源，健全社区服务体系。做好社区居委会换届工作。健全有效预防和化解社会矛盾的体制机制，完善人民调解、行政调解、仲裁调解、司法调解联动工作体系。三是加强和改进人口服务管理。创新人口调控手段，合理控制人口规模，优化人口结构，提高人口素质。统筹考虑财政承受能力、城市承载力，稳妥有序推进户籍制度改革，促进基本公共服务均等化。四是提升城市公共安全水平。落实城市公共安全白皮书，完善城市公共安全体系。建设平安深圳，强化社会治安综合治理，加大对网络违法犯罪活动的打击力度，刑事治安总警情继续下降。加大安全生产监管力度，健全隐患排查治理和安全预防控制体系，强化对城中村、“三小场所”、商贸市场、高层建筑等重点区域消防安全隐患排查整治，强化建筑工程安全监管，坚决遏制重特大安全事故。推进重点片区防洪蓄排工程建设。完善气象高级别预警全网发布机制。

　　（十）着力“两个转变”，建设为民务实清廉政府。做好今年各项工作，必须切实加强政府自身建设，抓好职能和作风“两个转变”，不断提高服务质量和水平，加快推进政府治理能力现代化。一是着力推进政府职能转变。最大限度减少直接管理、微观干预和事前审批准入，强化间接管理、宏观调控和事中事后监管服务。加强发展战略、规划、政策、标准等制定和实施，切实履行好公共服务、市场监管、社会管理、环境保护等职能，更好地弥补市场失灵。二是着力推进政府作风转变。严格落实中央八项规定和省、市实施意见，切实抓好群众路线教育实践活动整改落实，健全反对“四风”长效机制。坚持以人为本、服务民生，扎扎实实为群众办实事、办好事。大力改进文风会风，落实领导干部深入基层调研、挂点联系服务企业制度和重大项目分级协调机制，加快建设服务型政府。三是坚持依法行政。认真落实人大及其常委会各项决议决定，健全重大事项向人大及其常委会报告和向政协通报制度，积极主动接受市人大及其常委会的法律监督、工作监督和市政协的民主监督，认真听取民主党派、工商联、无党派人士和人民团体的意见，拓展协商民主形式。加强同人大代表和政协委员联系。认真办好建议提案，完善办理机制，提高办理质量。深入推进政府信息公开，充分发挥社会监督和舆论监督作用。用法治思维和方式履行政府职能，加强法制宣传教育，有效推进法律实施，加快建设一流法治城市。四是狠抓廉政建设。始终保持反腐败高压态势，切实加强反腐倡廉制度建设，严格执行廉政准则，强化行政监察和审计监督，加强廉政风险防控，推进廉洁城市建设。严格落实党政机关厉行节约反对浪费条例，强化财政资金监管，严格控制行政经费，实现“三公”经费总额再下降8%以上。同时，全面启动“十三五”国民经济和社会发展规划研究制定工作，描绘特区新一轮改革发展宏伟蓝图。

　　各位代表，新的一年开启新的希望，新的征程承载新的梦想。让我们高举中国特色社会主义伟大旗帜，在党中央国务院、省委省政府和市委的坚强领导下，进一步解放思想、锐意进取、真抓实干，奋力开创经济特区更加美好的未来，在新一轮改革开放中谱写中国梦的深圳篇章！
